# The limitations for expression recognition in computer vision introduced by facial masks

**DOI:** 10.1007/s11042-022-13559-8

**Published:** 2022-08-13

**Authors:** Andrea Francesco Abate, Lucia Cimmino, Bogdan-Costel Mocanu, Fabio Narducci, Florin Pop

**Affiliations:** 1 Department of Computer Science, University of Salerno, via Giovanni Paolo II, 132, Fisciano, 8484 Salerno Italy; 2 Department of System Engineering Faculty of Automatic Control and Computers, University Politehnica of Bucharest, Splaiul Independentei 313, Bucharest, RO-060042 Romania

**Keywords:** Expression recognition, Masked face analysis, Deep learning

## Abstract

Facial Expression recognition is a computer vision problem that took relevant benefit from the research in deep learning. Recent deep neural networks achieved superior results, demonstrating the feasibility of recognizing the expression of a user from a single picture or a video recording the face dynamics. Research studies reveal that the most discriminating portions of the face surfaces that contribute to the recognition of facial expressions are located on the mouth and the eyes. The restrictions for COVID pandemic reasons have also revealed that state-of-the-art solutions for the analysis of the face can severely fail due to the occlusions of using the facial masks. This study explores to what extend expression recognition can deal with occluded faces in presence of masks. To a fairer comparison, the analysis is performed in different occluded scenarios to effectively assess if the facial masks can really imply a decrease in the recognition accuracy. The experiments performed on two public datasets show that some famous top deep classifiers expose a significant reduction in accuracy in presence of masks up to half of the accuracy achieved in non-occluded conditions. Moreover, a relevant decrease in performance is also reported also in the case of occluded eyes but the overall drop in performance is not as severe as in presence of the facial masks, thus confirming that, like happens for face biometric recognition, occluded faces by facial mask still represent a challenging limitation for computer vision solutions.

## Introduction

Emotions understanding plays a key role in everyday people’s lives and interactions. Recognizing what a person is experiencing in a certain moment is part of the so-called emotional intelligence. Emotional intelligence is defined as the capacity of an individual to notice, discriminate, and identify other people emotions, as well as categorize them effectively. Similarly, for an automated system, being able to understand how the user feels while performing an interaction through a graphic user interface (GUI), for example, can allow the system itself to make the user experience more pleasant and improve the human-computer interaction quality. The most common way of recognizing emotions is through the interpretation of facial expressions. Normally, people show their emotional state, such as sadness, happiness, or anger, through facial expressions. The recognition of facial expressions is part of the AFEA (Automatic Facial Expression Analysis) systems, which aim to automatically analyse and recognize facial expressions using visual information, movements, and changes in facial features [31]. Affective computing and Sentiment analysis [22] are other two emerging fields that make emotion understanding and expressing, key arguments of the current human-computer studies. A wide range of prospective applications in many disciplines, such as Virtual Reality, smart surveillance systems, perceptual interfaces, and so on, are driving this growing interest in emotion understanding. Affective computing, as well as sentiment analysis and facial expression recognition systems, involve multidisciplinary fields such as cognitive, psychology, and computer sciences [30]. Most of the existing emotion recognition systems use artificial intelligence, in particular, deep learning models that analyse facial images and classify the emotion expressed in the pictures with good accuracy.

### FER and COVID-19

Typically, Facial Expression Recognition (FER) systems work on the features of the whole face for a person’s emotion identification. With the COVID-19 pandemic this assumption seems to be not obvious: the use of PPEs (Personal Proactive Equipment) and the enforcement of wearing a facial mask to protect people from the infection, raised a significant issue for all these systems that use the human face as a principal biometric trait for the analysis. The use of masks makes the emotion recognition problem even more challenging. Facial masks hide almost half of the human face, covering the area from the nose to the chin. In such a scenario the lower part of the face is completely unavailable for emotion understanding and a significant part of the information is lost. Nowadays the emotion recognition algorithms can efficiently identify seven basic emotions (Angry, Disgust, Fear, Happy, Neutral, Sad, and Surprise) achieving good recognition performance, but what happens if these algorithms are applied on masked faces?

**Fig. 1:**
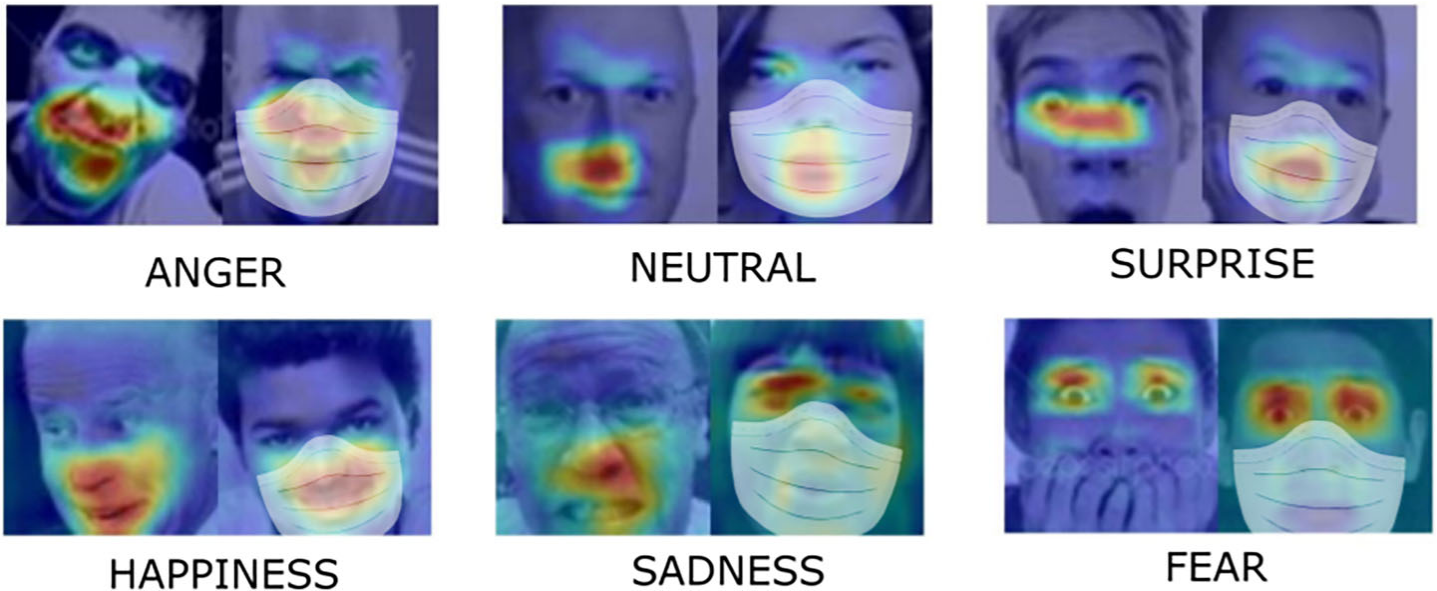
Most of the significant portion of the face that is useful for facial expression is on the region of the mouth. The presence of a facial mask can represent a relevant limitation in expression recognition

The motivations beyond this research can be found in the above question, posed in a particular historical period of Pandemic. Furthermore, a study conducted by [15] analyse the contribution of different face areas to different emotions. Their results show that the mouth has the highest classification accuracy since the mouth area seems to contain a lot of information about facial emotion. In particular, for the Neutral, Happiness, and Anger, the mouth reaches the highest performance among the other basic facial areas such as the nose or eyes. Fear emotion, instead, is the only one in which eyes lead to the least confusion. In Fig. 1 the heatmaps highlight the areas most affected in the emotion recognition process for each of the six basic emotions, at the same time the masks’ overlapping allows to visually understand which facial information is lost if an individual wears a mask.^1^

In this work, an analysis of the behaviour of the top FER methods existing in literature when the face analysed is covered by a mask is presented. Three top methods, with available code, are considered: ResidualMaskingNetwork [18], Facial-Expression-Recognition-with-CNNs [7] and Amend-Representation-Module [27]. Given the best configuration of each method, four experimental settings were applied:

1. Scenario 1: unmasked faces for training and masked faces for test.
2. Scenario 2: both training and test sets with masks.
3. Scenario 3: faces with occluded eyes for test and original face images for training.
4. Scenario 4: both training and test sets are images with occluded eyes.

In the next section, a brief description of the related works is reported. The rest of the paper is organised as follows: Section 3 presents the three top methods applied for the experimentation, Section 4 shows the results and discussion involving the four experimental settings; while Section 5 draws the conclusions of this work.

## Related works

Ekman and Friesen [6] pointed out that facial expression of emotions can be considered universal, in particular, they defined six basic emotions: Anger, Afraid, Happiness, Sadness, Disgust, and Surprise. A seventh emotion, the Neutral one, was added later, modern and most accurate FER systems tend to recognize all the seven emotions, sometimes the neutral emotion is used as a sort of benchmark to test the system performances. A first attempt to automatically study expressions was presented by Sown [29], who analysed facial expressions by tracing the movement of 20 points located on the face, in image sequences. Since then, much progress has been made in building systems that aim to effectively recognize facial expressions.

Facial Expression Recognition systems can be classified through two main criteria: i) the use of videos or static images through machine learning solutions and ii) the use of deep learning methods versus the traditional approach based on handcrafted features.

### Image and video analysis through machine learning

Static expression recognition is referred to the use of facial images, which show a specific expression in the picture, to extract a set of static features [2]. The video-based (dynamic) FER uses spatio-temporal information to analyse the dynamics of the expressions captured in a video sequence [17]. The additional temporal information allows dynamic FER to achieve better performance than static FER. However, some drawbacks have to be considered: significant differences can be present in the extracted dynamic features in terms of the duration of transition as well as in terms of features characteristics of the expression depending on the person’s physiognomy. Dynamic features may also be subject to a normalization of the frames that leads to a loss of temporal information [10]. Since the methods tested on the occluded images use solely static facial features, only image-based works are considered. Several conventional approaches have been studied to solve automatic emotion recognition problem. They are characterized by a features extraction step in which geometric and/or appearance features are extracted from facial images, and then a classification model is applied to classify the expressions. Ali et al. [1] use Empirical Mode Decomposition (EMD) techniques for facial emotion recognition, applying 3D reduction algorithms and using reduced features to feed Support Vector Machine (SVM), k-Nearest Neighbour (k-NN), and Radial Basis Function (ELM-RBF) classifiers for the classification of seven basic facial emotions. Two types of geometric features are extracted in the approach by Jung et al. [9]. They use the position of 52 facial points to calculate distances between points and angles. For the classification, AdaBoost with dynamic time warping and SVM on the boosted feature vectors are applied. A depth information-based approach is proposed in Lee and Lee [12]. Lee and Lee extract 10 geometric points from images captured through structured-light type RGB-D camera. As a result of their study, the 2D image-based method has higher classification performance than the 3D depth information-based method.

### Deep learning approaches

In recent years, the trend of deep learning methods has *particularly* involved the field of computer vision and image understanding [21]. The main advantage of Convolutional Neural Networks (CNNs) is a complete loss or a high reduction of the pre-processing and feature extraction phases. This means that the CNNs learn directly from the input images [8]. Moreover, the application of CNNs techniques for image classification tasks has achieved considerable results. Most deep-learning-based methods approaches have directly adapted a CNN for expression recognition. Pranav et al. [25], for example, proposes a new Deep Convolutional Neural Network (DCNN) model that classifies 5 human facial emotions. The model architecture is composed of two convolution layers with dropouts after each convolution layer. Ng et al. [23] apply a transfer learning technique for deep CNN architectures with two-stage supervised fine-tuning. They use AlexNet [11] and the publicly available VGG-CNN-M-2048 [5] models, which are pre-trained on imageNet [26] dataset. One of the major drawbacks/concerns of using the CNN model is the availability of a large image dataset, plus in uncontrolled environments, the facial images can suffer from different forms of noise such as blur, occlusion, illumination variations, etc. Trying to deal with these challenging points of facial expression recognition, Umer et al. [32] propose an efficient deep learning-based framework that uses a convolutional neural networks (CNN) architecture to extract more discriminant features. A novel Data augmentation technique has been applied to enhance the training capability of the CNN model, obtaining one of the best performances among the existing CNN-based FER systems. In this direction, three machine learning contests were held in 2013 as a part of the ICML workshop “Challenges in Representation Learning”. The workshop aimed at exploring the latest developments in representation learning. One of the challenges involved the facial expression recognition task. The participants were invited to design the best system for recognizing which emotion is being expressed in a photo of a human face Goodfellow et al. [7]. Residual Masking Network [19] results to achieve the best performances in the contest. Pham et.al. use a segmentation network to refine feature maps, so the network is able to focus on the relevant information and make correct decisions. Good performance is also reported by Shi and Zhu [27] with their novel architecture: Amending Representation Module (ARM). ARM efficiently affords the facial expression recognition challenges, boosting the performance of FER remarkably. in fact, validation accuracy is around the 90%. In this work three top static frame-based FER methods, which use deep learning, are analysed and compared, namely the aforementioned ARM module, and Residual Masking Network and Facial-Expression-Recognition-with-CNNs from the ICML workshop “Challenges in Representation Learning” Goodfellow et al. [7]. In recent times the outbreak of the COVID-19 disease has stimulated research into different topics associated with the pandemic: from automatic COVID-19 disease detection, as explored by [4], to the role of IoT technologies in pandemic control [3], to the issues caused by the facial masks in biometric face recognition [14].

## Analysed methodology

A set of three top deep learning-based algorithms for recognizing emotions in static images has been chosen among the systems currently presented in the literature. The selection of the methods involved both the performances and the availability of the code on the web. More specifically, the code for each method is available on GitHub in the following repository:

1. Residual Masking Network Luan et al. [20]
2. Facial-Expression-Recognition-with-CNNs Nischal [24].
3. Amending Representation Module (ARM) Shi and Zhu [28]

1. **Residual Masking Network** is presented in Luan et al. [19] is a novel facial expression recognition model that uses a Masking Idea to boost the recognition performance. This masking idea is based on the fact that a localization network can contribute to the optimization of tensors by generating importance weights, allowing the learning process to focus on what it considers necessary. The network is composed of four Residual Masking Blocks. Each of these blocks contains a Residual layer and a Masking Block. The input image goes in a first 3x3 convolutional layer with stride 2 and then passes to a 2x2 max-pooling layer. The next four Residual Masking Blocks transform the obtained convolutional maps into four other features maps of four spatial sizes, namely 56 × 56, 28 × 28, 14 × 14, and 7×7. To produce outputs corresponding to the seven basic emotions, the network includes an average pooling layer and a 7-way fully-connected layer with a softmax function.
  A representation in brief of the Residual Masking Network is shown in Table 1
2. **Facial-Expression-Recognition-with-CNNs** is a publicly available Convolutional Neural Network built in Keras and Tensorflow. The repository is under MIT license Nischal [24]. The model architecture is organized as follows:
  *(i)* Convolutional blocks (ConvBlock): four blocks of sequential 2D Convolutional, batch normalization, activation, maxpooling2D, and dropout layers transform the input shape from (48,48,64) to (6,6,512); *(ii)* a flatten layer is applied and the data dimension is reduced from two dimensional to one dimensional data shape. *(iii)* Fully connected blocks (FullyConnBlock) are other two blocks of sequential, dense, batch normalization, activation, and dropout layers, applied to prepare the tensor for the last dense layer whose size produces outputs corresponding to the seven emotions. Table 2 shows the network architecture: for each block, the output and some configuration details are reported. The symbols *k* and *s* represent the Kernel of Conv2D layers and MaxPooling filter size, respectively. The model is designed to recognize the seven basic emotions: Angry, Disgust, Fear, Happy, Sad, Surprise, and Neutral.
3. **Amending Representation Module** is another novel Convolutional Neural Network Architecture proposed in Shi and Zhu [27]. It consists of three key blocks: Feature Arrangement (FA) block, Dealbino (DA) block, and Sharing Affinity (SA) block. The FA block serves as an add-on to the DA block, enhancing its functionality. By employing convolution, the latter achieves the weight distribution of features. The network architecture is shown in Table 3.

**Table 1:** The network architecture of Residual Masking Network model

Layer	Output	Details
*Conv*_1_	64*@*112 × 112	*k* = 3
*MaxPooling*	*@*56 × 56	*s* = 2*x*2
*ResMaskingBlock*_1_	64*@*56 × 56	*RL*_1_,*MB*_1_
*ResMaskingBlock*_2_	128*@*28 × 28	*RL*_2_,*MB*_2_
*ResMaskingBlock*_3_	256*@*14 × 14	*RL*_3_,*MB*_3_
*ResMaskingBlock*_4_	512*@*7 × 7	*RL*_4_,*MB*_4_
*AveragePooling*	512*@*1 × 1	
*FC*,*SoftMax*	(*None*, 7)

**Table 2:** The network architecture of Facial-Expression-Recognition-with-CNNs model

Layer	Output	Details
*ConvBlock*_1_	64*@*24 × 24	*k* = 3; *s* = 2*x*2
*ConvBlock*_2_	64*@*12 × 12	*k* = 3; *s* = 2*x*2
*ConvBlock*_3_	64*@*6 × 6	*k* = 3; *s* = 2*x*2
*ConvBlock*_4_	64*@*3 × 3	*k* = 3; *s* = 2*x*2
*Flattern*	(*None*, 4608)	
*FullyConnBlock*_1_	(*None*, 256)	
*FullyConnBlock*_2_	(*None*, 512)	
*Dense*_3_	(*None*, 7); *units* = 7

**Table 3:** The network architecture of Amending Representation Module

Layer	Output	Details
*ResNet* − 18	(*None*, 512, 7, 7)	
*Arrangement*	(*None*, 2, 112, 112)	
*De* − *Albino*	(*None*, 2, 11, 11)	*k* = 3; *s* = 8*x*8
*BatchNorm*	(*None*, 2, 11, 11)	
*Mean*	(*None*, 11, 11)	
*Affinity*	(*None*, 11, 11)	
*FullyConnected*	(*None*, 7)
  The weight with a larger degree of albino erosion is generally lower, and vice versa. The last SA block separates facial features into two parts to simplify representation learning. Experiments using publicly available benchmarks show that this model significantly improves FER’s performance. In fact, among the analyzed methods, the Amend Representation model is the one achieving the best accuracy.

Residual Masking Network and FER-with-CNNs are trained on to Facial Expression Recognition 2013 Database (FER2013) dataset, Amending Representation Module, instead, is trained on Real-world Affective Faces Database (RAF-DB). The aim of this work is to test the models on faces occluded by facial masks, for this reason, and for a fair comparison of the performance achieved by each method, different versions of the aforementioned datasets were used for benchmarking: FER2013 masked and FER2013 eye-occluded, RAF-DB masked and RAF-DB eye-occluded.

### Datasets

#### Facial expression recognition 2013 database

**Fig. 2:**
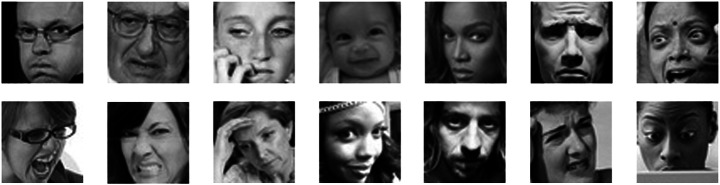
Samples of FER2013 Dataset

Facial Expression Recognition 2013 Database (FER2013) Goodfellow et al. [7] is composed of 48×48 pixel images of faces in grayscale. The faces are centered in the frame and are arranged in a way that faces uniformly cover the same amount of space in each image. An overview of the dataset is provided in Fig. 2. The images are divided into seven folders, one for each of the seven basic emotions (0=Angry, 1=Disgust, 2=Fear, 3=Happy, 4=Sad, 5=Surprise, 6=Neutral). There are 28,709 samples in the training set, while the test set consists of 3,589 examples, for a total of 32,298 facial images.

#### Real-world affective faces database

**Fig. 3:**
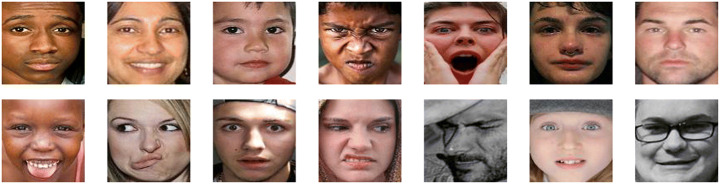
Samples of RAF-DB Dataset

The Real-world Affective Faces Database (RAF-DB) Li et al. [13] is a large-scale face expression database that contains around 30k facial images collected from the internet. Based on the crowd sourcing annotation, RAF-DB includes 29,672 real-world 100×100 pixel RGB facial images: 3,068 for the test, the rest for the training. A .txt file contains the expression label for each image: 1=Surprise, 2=Fear, 3=Disgust, 4=Happy, 5=Sad, 6=Angry, 7=Neutral. In Fig. 3 a subset of facial images from the RAF-DB dataset is shown.

#### Masked and eye-occluded datasets

A version of masked and eye-occluded datasets to perform experiments for the different Scenarios is presented in this subsection. For Scenarios 1 and 2 fake masks are applied to each face image in the datasets. The two datasets were synthetically masked using an algorithm that masks facial photos by overlaying masks on a detected face.^2^ A simple black occlusion bar is placed on the periocular region of the faces in the databases for Scenarios 3 and 4.

**Fig. 4:**
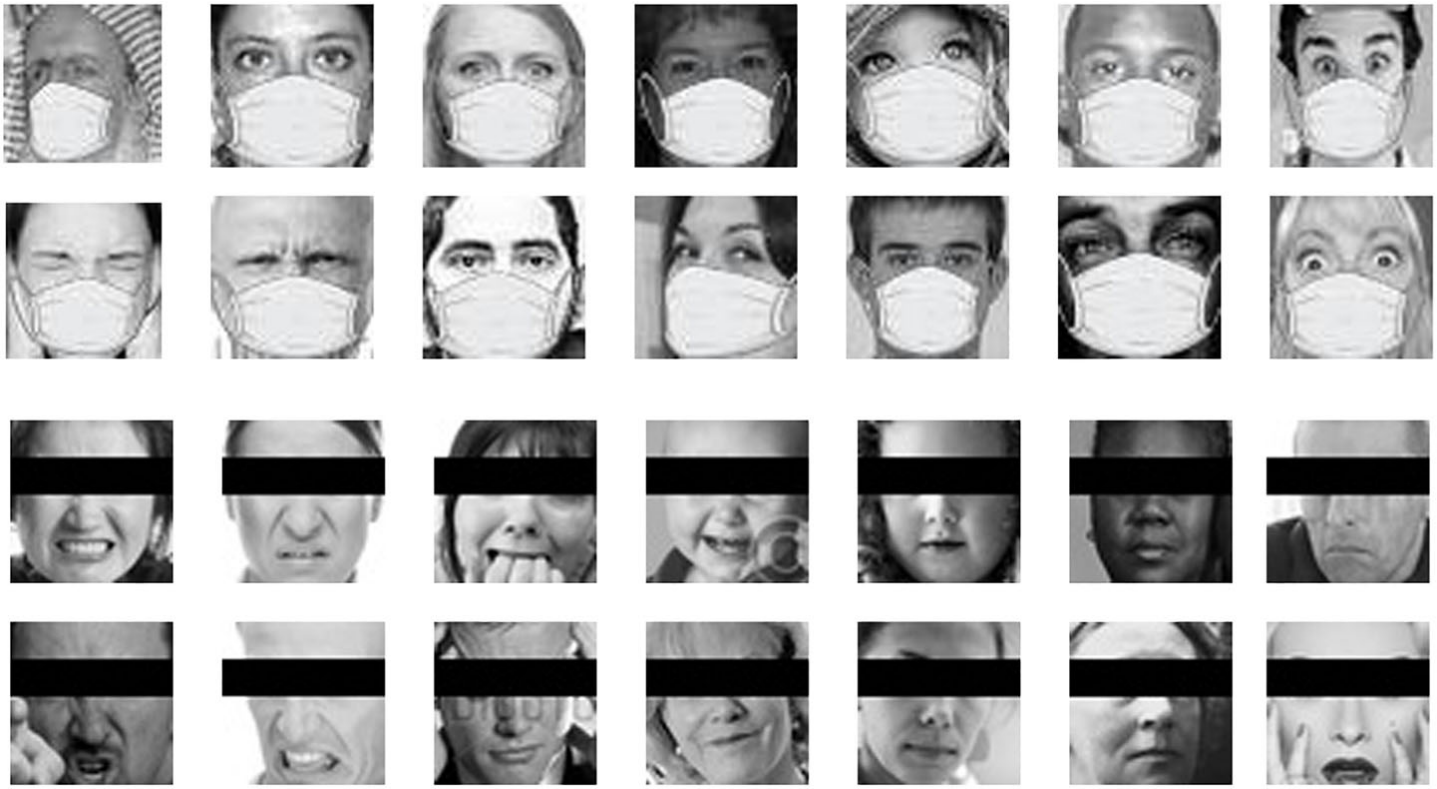
Samples of FER2013 Masked and eye-occluded Dataset

**Fig. 5:**
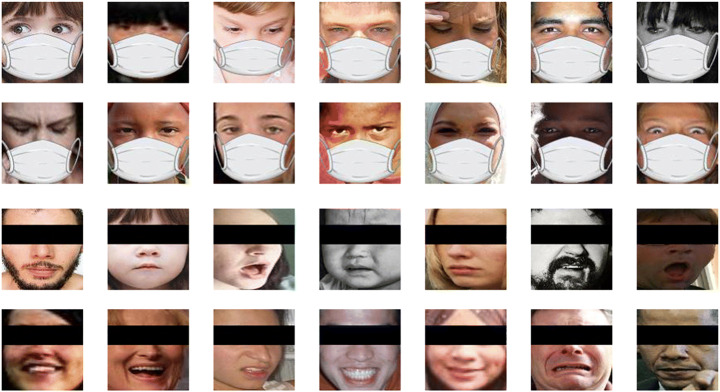
Samples of RAF-DB Masked and eye-occluded Dataset

The number of training and test examples for both datasets is unchanged. In Figs. 4 and 5, some samples of FEER2013 Masked and eye-occluded, and RAF-DB masked and eye-occluded is shown.

## Results and discussion

Starting from the best configuration of each model described in Section 3, four experimental settings were considered to test and compare the performances of masked faces:

1. Scenario 1: in this first setup, unmasked faces were used for training and masked faces for the test.
2. Scenario 2: both training and test sets consist of masked faces.
3. Scenario 3: mirroring the first Scenario, this configuration tests the model trained on the original dataset using eye-occluded images.
4. Scenario 4: eye-occluded images are used for the training and the test phases.

The results obtained applying each experimental configuration to the three top models are summarised in Table 4.

**Table 4:** Accuracy, precision, recall and F1-score achieved by the methods tested in the four scenarios and compared to the results achieved in the original datasets without masks and eye occlusions

Model	Dataset	Metric	Original	Scenario1	Scenario2	Scenario3	Scenario4
Residual masking	FER2013	*accuracy*	74.14%	32.97%	62.03%	54.50%	68.51%
network [18]		*precision*	74.36%	42.78%	64.12%	54.71%	68.61%
		*recall*	74.14%	32.97%	62.03%	54.50%	68.51%
		*F1-score*	74.25%	37.24%	63.06%	54.60%	68.56%
FER CNNs [7]	FER2013	*accuracy*	62.90%	32.10%	54.20%	45.58%	56.80%
		*precision*	63.10%	33.24%	59.09%	48.09%	57.44%
		*recall*	62.90%	32.10%	54.20%	45.58%	56.80%
		*F1-score*	63.00%	32.66%	56.54%	46.80%	57.12%
Amend-Representation	RAF-DB	*accuracy*	90.42%	45.45%	82.30%	74.25%	84.32%
Module [27]		*precision*	90.26%	56.16%	81.75%	74.25%	84.03%
		*recall*	90.42%	45.45%	82.30%	74.56%	84.32%
		*F1-score*	90.34%	50.24%	82.02%	74.40%	84.17%

For Scenario 1, a drop in the performances is reported for all compared methods: Residual Masking Network goes from an accuracy of 74,14% to 35,65% and a similar decrease can be observed with the FER CNNs model too. The worst-case scenario, which displays the highest decline, is the Amend-Representation Module. Despite having the best performance, it shows a greater decrease than the first two methods, specifically it goes from an originally reported accuracy of 90,42% to 45,45% using a masked dataset for the test phase. This means that a method designed to recognize emotions from a non-occluded face significantly fails if it is tested on masked faces. Performance improves when models are trained on masked datasets. An expected result since the models have seen masked faces also in the training examples. Anyway, in this second Scenario, the accuracy also shows a non-negligible average decrease of 10%. In order to verify if the loss of the mouth was truly significant with respect to any other obstruction, in Scenarios 3 and 4 images with periocular occlusions were taken into consideration. In this case, the greatest decrease can be observed in the scenario where only the test set is eye-occluded (Scenario 3). Anyway, this loss in performance appears less marked than the one in Scenario 1. The average decrease, in fact, results to be 28% compared to the 40% observed in the first Scenario against the original accuracy. Although there is a significant difference in performance between Scenario 1 and Scenario 3, the accuracy scores for Scenario 2 and 4 are quite similar. The Scenario involving mouth occlusions (Scenario 2) has a higher drop, however, there is a difference of roughly 12% between the accuracy of Scenario 2 and Scenario 4. The latter scenario shows the smallest drop in accuracy which averages around 5% compared to the original results.

**Fig. 6:**
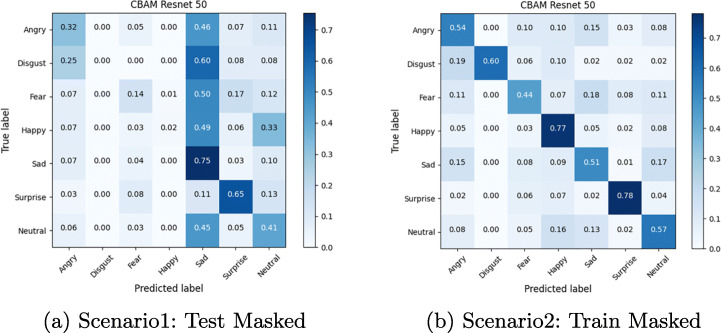
Confusion Matrix of ResMasking Module on Masked FER2013 dataset

**Fig. 7:**
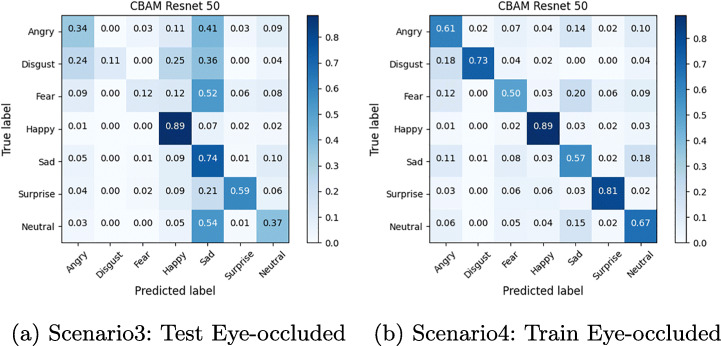
Confusion Matrix of ResMasking on Eye-occluded FER2013 dataset

**Fig. 8:**
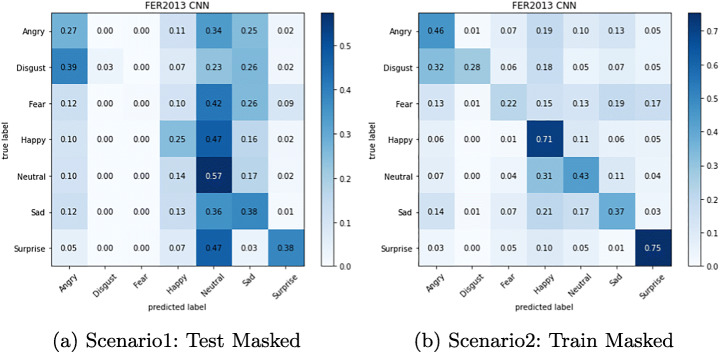
Confusion Matrix of FER-CNNs Module on Masked FER2013 dataset

**Fig. 9:**
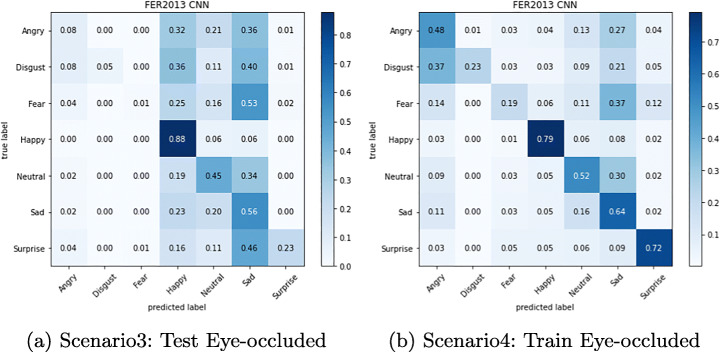
Confusion Matrix of FER-CNNs Module on Eye-occluded FER2013 dataset

**Fig. 10:**
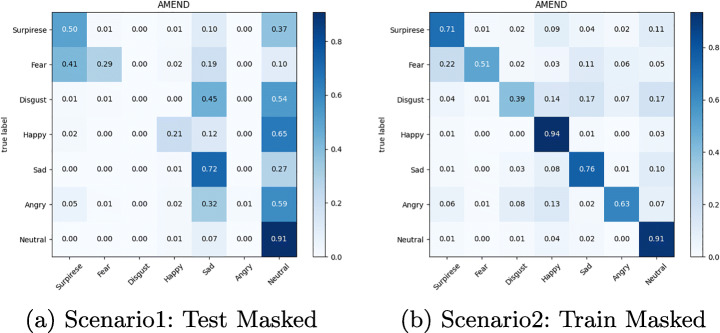
Confusion Matrix of AR Module on Masked RAF-DB dataset

**Fig. 11:**
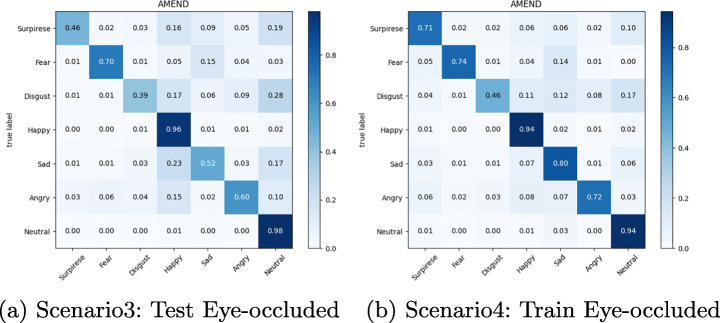
Confusion Matrix of AR Module on Eye-occluded RAF-DB dataset

The confusion matrix depicted in Figures from Figs. 6, 7, 8, 9, 10 and 11 reflect the situation described earlier. In addition, they provide information on emotions that are better recognized than others.

In particular, it can be observed that the best recognized emotion is the Happy one for almost every model in each Scenario. ResMasking and AR modules are able to better discriminate also the Neutral emotion. The only case in which Neutral and Surprise emotions present a better behavior than the Happy one is reported in Fig. 8a which represents the Scenario 1 confusion matrix for the FER-CNN Model. Another difference that can be seen between the FER-CNNS module and the other two is that the Surprise emotion in Scenarios 2 and 4 (Figs. 8b and 9b respectively) is better than the Neutral.

Experimental results show an expected consideration about the reduction in terms of accuracy in all scenarios that introduce an occlusion compared to the level of performances achieved in full visible face. On the other hand, it can be observed that the occlusions of the periocular areas are not as severe as those due to facial masks. In presence of facial masks, the deep classification models register a decrease in accuracy during the testing stage up to half of the performance achieved in normal operative conditions. Conversely, a similar dramatic trend is not reported when the models are exposed to the difficulties of working with occlusions of the periocular area. In such a last case, the overall level of performance does not compare with ideal conditions but the failure cases as such that the mean accuracy states about -10%.

## Conclusions

In computer vision, emotion recognition is the process of identifying human emotions from images. Differently for other well-assessed approaches to analysis of the face like biometric recognition, the recognition of the facial expression is not a trivial task. People learn to classify the expressions since childhood and they can vary widely in the accuracy at recognizing the emotions of others according to the experience and nationality. The use of digital solutions for emotion recognition is a relatively emerging research area. Deep learning approaches have been demonstrated to achieve encouraging levels of accuracy in ideal conditions and even in-the-wild scenarios. On the other hand, COVID pandemic restrictions questioned all computer visions algorithms for face analysis for a wide range of purposes due to the presence of occluding facial masks. Even though research studies demonstrated that the periocular region can help the classification of some expressions, e.g., surprise, fear, disgust, and anger, many others are strongly dependent on the analysis of labial area.

In this work, the attention is focused on the analysis of state-of-the-art solutions for expression recognition from still images in presence of facial masks. The objective is to discuss how feasibly the most performing algorithms can be retrained to take into account the presence of the occlusions introduced by the facial masks and the level of performance that can achieve in this challenging acquisition condition. For a fairer comparative analysis, the results obtained in presence of the facial masks are analysed against the occlusion of the periocular region. These considerations led to exploring different experimental scenarios where the occlusions are interleaved with normal/ideal conditions starting from two public datasets for expression recognition.

The results reported in this study reveal that recent pandemic restrictions are significantly affecting all those application scenarios where the analysis of the human face is useful to enable advanced human computer interaction. For example, human-robot interaction is a typical case where the recognition of human emotion by the robot can improve the interaction among the parties. Also in public indoor areas, expression recognition can be helpful for marketing and advertisement. Since the COVID pandemic is demonstrating that our life has been significantly changed and that facial masks seem to be mandatory for a long time, longer than the current expectations, face analysis must take into consideration this higher level of complexity in the problem of extracting information from the human’s face. We believe that the results reported in our study are useful to point attention to the problem and to consider it as a fruitful emerging field of research over this time.

## Footnotes

1 Original figure from Lian et al. [16] which has been modified with virtual masks in this work.

2 https://github.com/Prodesire/face-mask
